# Substituted Piperazines as Novel Potential Radioprotective Agents

**DOI:** 10.3390/molecules25030532

**Published:** 2020-01-25

**Authors:** Alzbeta Filipova, Jan Marek, Radim Havelek, Jaroslav Pejchal, Marcela Jelicova, Jana Cizkova, Martina Majorosova, Lubica Muckova, Tomas Kucera, Lukas Prchal, Miroslav Psotka, Natalie Zivna, Darja Koutova, Zuzana Sinkorova, Martina Rezacova, Ales Tichy

**Affiliations:** 1 Department of Radiobiology, Faculty of Military Health Sciences, University of Defence in Brno, Trebesska 1575, 500 01 Hradec Kralove, Czech Republic; alzbeta.filipova@unob.cz (A.F.); Marcela.Jelicova@unob.cz (M.J.); Jana.Cizkova@unob.cz (J.C.); Zuzana.Sinkorova@unob.cz (Z.S.); 2 Biomedical Research Center, University Hospital Hradec Kralove, Sokolska 581, 500 05 Hradec Kralove, Czech Republic; marekjjja@gmail.com (J.M.); lukas.prchal@fnhk.cz (L.P.); miroslav.psotka@fnhk.cz (M.P.); natalie.zivna@natur.cuni.cz (N.Z.); 3 Department of Medical Biochemistry, Faculty of Medicine in Hradec Kralove, Charles University, Simkova 870, 500 03 Hradec Kralove, Czech Republic; havelekr@lfhk.cuni.cz (R.H.); SeifrtovaM@lfhk.cuni.cz (M.M.); koutova.darja@lfhk.cuni.cz (D.K.); rezacovaM@lfhk.cuni.cz (M.R.); 4 Department of Toxicology and Military Pharmacy, Faculty of Military Health Sciences, University of Defence in Brno, Trebesska 1575, 500 01 Hradec Kralove, Czech Republic; jaroslav.pejchal@unob.cz (J.P.); Lubica.Muckova@unob.cz (L.M.); Tomas.Kucera@unob.cz (T.K.)

**Keywords:** piperazine, radiation-protective agents, cytotoxicity, maximum tolerated dose, synthesis de novo

## Abstract

The increasing risk of radiation exposure underlines the need for novel radioprotective agents. Hence, a series of novel 1-(2-hydroxyethyl)piperazine derivatives were designed and synthesized. Some of the compounds protected human cells against radiation-induced apoptosis and exhibited low cytotoxicity. Compared to the previous series of piperazine derivatives, compound **8** exhibited a radioprotective effect on cell survival in vitro and low toxicity in vivo. It also enhanced the survival of mice 30 days after whole-body irradiation (although this increase was not statistically significant). Taken together, our in vitro and in vivo data indicate that some of our compounds are valuable for further research as potential radioprotectors.

## 1. Introduction

Ionizing radiation (IR) was discovered in the 19th century; since then, its use has had a dramatic impact on medicine, space travel, research, and the energy industry. Unfortunately, the catastrophic effects of IR have also been exploited by the development of nuclear weapons; moreover, a number of accidents have occurred at nuclear power reactors resulting in ecological disasters and loss of lives [1,2,3].

Exposure to IR is associated with cell death, genetic mutations, and carcinogenesis [4]. It induces DNA damage in the form of double-strand DNA breaks, which is considered the underlying mechanism of the resulting cell death: apoptosis.

Apoptosis is a very complex process, which is tightly controlled in mammalian cells [5]. Its multi-level regulation has been the subject of medical research for a long time because some pathologies (such as cancer and autoimmune and neurodegenerative disease) are closely associated with the dysregulation of programmed cell death. Above that, individual regulatory systems provide suitable targets for novel compounds with desirable pharmacological potential [6,7,8,9]. The testing of thousands of synthetic and natural compounds has consequently led to the discovery of novel radioprotectants or mitigators. Unfortunately, these compounds can often be insoluble or cytotoxic, with low tolerance, or with very low circulating half-life. As a result, their clinical application is rather limited [10,11].

**Scheme 1:**
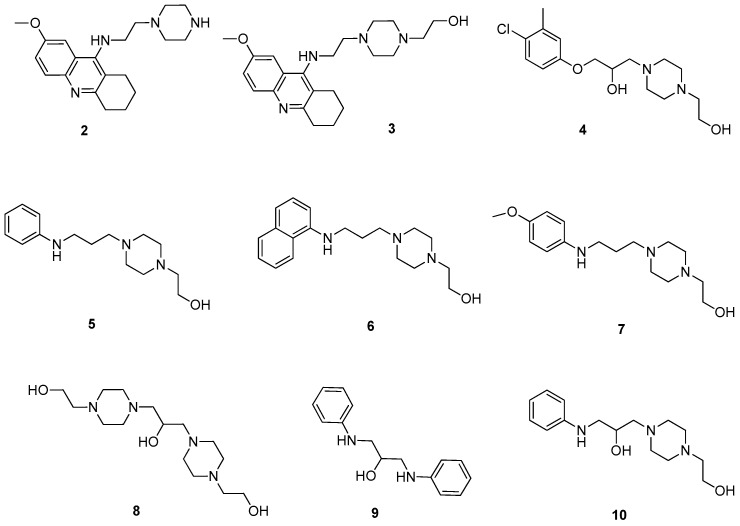
Synthesized compounds as bases with potential radioprotective properties.

The increasing risk of radiation exposure underlines the need for novel radioprotective agents [3,12,13]. However, there is very limited current literature concerned with small molecules as potential radioprotective agents. Based on previous studies, we have selected the 1-(2-hydroxyethyl)piperazine moiety (main part of compounds in Scheme 1, compound 1 in Scheme S4 in Supplementary Materials) as the essential component of the molecule [10,14]. Notably, some of our structures are already mentioned in the literature, but none has been reported in the context of radioprotection. Namely, compound **2** had already been prepared as a leading structure for a novel group of cholinesterase inhibitors [15]. Compound **3** was prepared afterwards as a novel structure according to the procedure for preparation of these inhibitors. Compound **4** was searched for in the PubChem and ZINC database [16], but no synthesis or biological data were found. The symmetrical molecule **9** consisting of two aniline residues had been prepared by Dains et al. in 1922 [17]. None of the other compounds had ever been prepared or tested before (**5**, **6**, **7**, **8**, and **10**).

Thus, the aim of this work was to investigate a group of 1-(2-hydroxyethyl)piperazine derivatives as potential affordable radioprotective agents. We focused on various modifications of the part of the molecule attached to the basic 1-(2-hydroxyethyl)piperazine moiety. We characterized the prepared compounds using nuclear magnetic resonance (NMR) and high-resolution mass spectrometry (HRMS), and evaluated their cytotoxicity and radioprotective properties in vitro and in vivo using human cell lines and an animal experimental model.

## 2. Results 

### 2.1. Chemistry

All the compounds developed in this study are outlined in Scheme 1. Compound **2** was synthesized as previously described; the results (yields, analytical data) are comparable to those formerly reported [15]. Compound **3** was prepared by the reaction of **2** with Hunig´s base and 2-bromoethanol, following also previously reported protocol (Scheme S1 in Supplementary Materials) [15]. The synthetic route for compound **4** is a two-step reaction. The first step is the reaction of 4-chloro-3-methylphenol (**4a**) with an excess of epibromohydrin under reflux for 2 h in the presence of piperidine, according to the process published in [14] (Scheme S2 in Supplementary Materials). This solvent-free reaction led to the preparation of intermediate **4b** in 70% yield (Scheme S2 in Supplementary Materials). **4b** was used for a further step without any purification. Accordingly, 1-(2-hydroxyethyl)piperazine was alkylated by **4b**, affording **4** in 79% yield (Scheme S2 in Supplementary Materials).

Compounds **5**, **6**, and **7** were synthesized similarly via a two-step reaction. The first step was the preparation of intermediates **5b**, **6b**, and **7b** (Scheme S3 in Supplementary Materials) by treatment appropriate aryl amines **5a**, **6a**, and **7a**, respectively, with the 1-bromo-3-chloropropane in acetonitrile under microwave conditions, all with rather poor yield around 30% (Scheme S3 in Supplementary Materials). **5b**, **6b**, and **7b** were used for the following step without any purification. The next step involved the alkylation of 1-(2-hydroxyethyl)piperazine (**1**) by the intermediates **5b**, **6b**, and **7b**, resulting in compounds **5**, **6**, and **7**, respectively, in 60–70% yields (Scheme S3 in Supplementary Materials). The procedure for the preparation of compound **8** was the reaction of 1-(2-hydroxyethyl)piperazine (**1**) with epibromohydrin in ethanol (Scheme S4 in Supplementary Materials). The yield of this one-step reaction was 27% due to the formation of side products. Symmetric molecule **9** was prepared using solvent-free reaction under microwave conditions in 25% yield (Scheme S5 in Supplementary Materials). Compound **10** was synthesized via a two-step reaction. The first step was the preparation of intermediate **10b** (Scheme S6 in Supplementary Materials). This intermediate was isolated during the procedure of preparation of **9** as the second expected product in a yield of 45%. The next step involved the alkylation of 1-(2-hydroxyethyl)piperazine (**1**) by the intermediate (**10b**), resulting in compound **10** in 90% yield (Scheme S6 in Supplementary Materials).

Finally, the compounds (**2**–**10**) (Table 1) were converted to their salts by treatment with hydrochloric acid in methanol. All the final compounds were characterized by their ^1^H- and ^13^C-NMR spectra and high-resolution mass spectrometry (HRMS). All analytical measurements confirmed the structure and purity of over 95% of the final compounds as determined by LC-MS analysis. All the spectral data of the compounds are provided in the Experimental section.

### 2.2. Molecular Docking with Anti-Apoptotic Protein Bcl-2

The docking calculations with ligand anti-apoptotic protein Bcl-2 (B-cell lymphoma 2) were performed for all of the investigated compounds. All of them showed hydrophobic interaction of the aromatic part (tetrahydroacridine, naphtyl, or phenyl moiety) with the hydrophilic pocket of the Bcl-2 protein (Leu134, Phe101, Tyr105, Phe109, Met112, Phe150 and Val130), except for compound **8**. The quarternary hydrogen of all compounds interacted with Asp108, except for compound **9**.

A hydrogen bond of uncharged piperazine nitrogen with Arg143 was observed frequently. Some of the compounds showed a hydrogen bond of aniline nitrogen with Asp108. Other interactions could not be judged as group characteristics but rather individual features.

**Figure 1:**
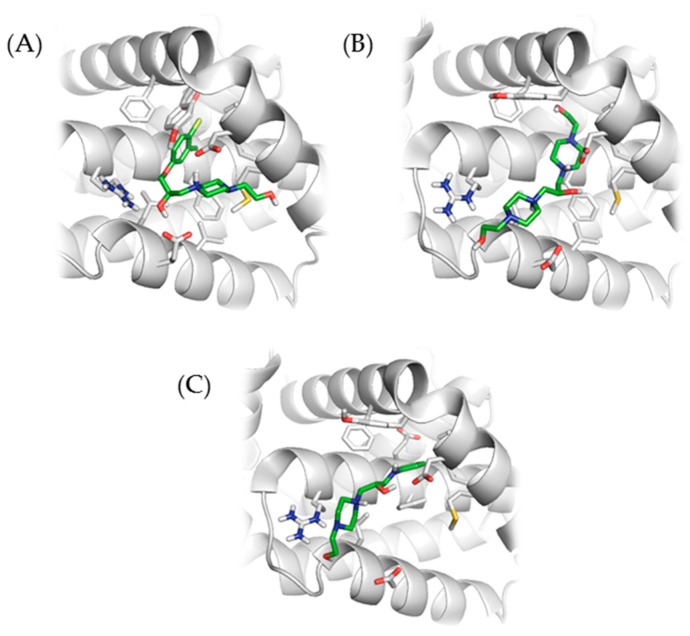
Interaction of selected compounds with Bcl-2 protein. (**A**) Interaction of compound 4 with Bcl-2: ionic interaction of protonated piperacilin nitrogen with Asp108, hydrogen bond of hydroxyl oxygen with Arg143, hydrophobic interaction of chlormethylphenyl part with the pocket Leu134, Phe101, Tyr105, Phe109, Met112, Phe150, and Val130. (**B**) Interaction of compound 8 with Bcl-2: ionic interaction of protonated piperacilin nitrogen with Asp108, hydrogen bonds Arg143 with hydroxyl oxygen. (**C**) Interaction of compound 10 with Bcl-2: hydrogen bonds of aniline group and of hydroxyl group with Asp108, and of piperacilin nitrogen and hydroxyl oxigen with Arg143, hydrophobic interaction of phenyl part with the pocket Leu134, Phe101, Tyr105, Phe109, Met112, Phe150, and Val130.

Interaction of compounds **4**, **8**, and **10** with Bcl-2 are shown in Figure 1.

### 2.3. In Vitro Cytotoxicity of Newly Synthesized Compounds ***2***, ***3***, ***4***, ***5***, ***6***, ***7***, ***8***, ***9***, and ***10***

In this study, the cytotoxic effects of nine new compounds (**2**, **3**, **4**, **5**, **6**, **7**, **8**, **9**, **10**) were tested on 10 human cell lines (nine cancer and one non-cancer cell line). The cell lines were treated with each compound at concentrations of 10 µM and 100 µM for 48 h. The proliferation capacity of the treated cells was determined by WST-1 test and compared against the proliferation rate of the untreated control cells (100%).

These experiments revealed that compound **2** (10 µM) was cytotoxic across all the 10 cell lines tested, as indicated by the percentage of viable cells which was less than 44%, especially in MOLT-4 cells. Accordingly, compound **2** was not tested at 100 µM. The remaining compounds had no evident cytotoxic effect on any of the tested cell lines.

Further experiments at 100 µM revealed a significantly lower number of living cells when compound **3** was used. The cytotoxic effect of this compound was detected in all the tested cell lines, decreasing cell viability by 70% and more, especially in MOLT-4 and MRC-5 cells. However, the remaining compounds (**4**, **5**, **6**, **7**, **8**, **9**, and **10**) had no significant cytotoxic effects on the studied cell lines (Table 2).

### 2.4. In Vitro Toxicity Determination of New Inhibitors

In further experiments, MTT (3-(4,5-di-methylthiazol-2-yl)-2,5-diphenyltetrazolium bromide) assay was employed to evaluate the toxicological indexes of the newly synthesized compounds, including IC_50_ (half-maximal inhibitory concentration) and MTC (maximum tolerated concentration). Since the assay is applicable only for adherent cells, the A-549 cell line was selected for these evaluations.

In A549 cells, the lowest IC_50_ was found for compound **2** (0.04 ± 0.002 mM). The tolerance of other compounds increased in the following order from **2** to **3**, **9**, **6**, **4**, **5**, **7**, **10**, and **8**, with corresponding IC_50_ values being 3.5-, 6.0-, 11.3-, 16.0-, 33.8-, 63.8-, 126.5-, and 625-fold higher than that for compound **2** (Table 3).

### 2.5. Pre-Treatment with Compounds ***4***, ***5***, ***6***, ***7***, ***8***, and ***10*** Reduced Radiation-Induced Apoptosis In Vitro

**Figure 2:**
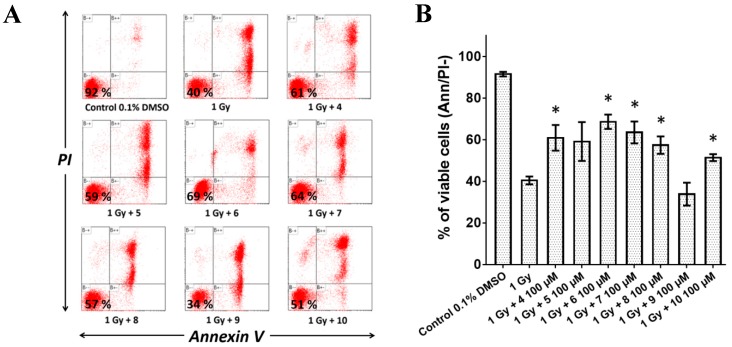
The viability of MOLT-4 cells after exposed to ionizing radiation (IR) alone or in combination with inhibitors at 100 μM. Viability of MOLT-4 cells was determined as the percentage of Annexin-V–Alexa Fluor^®^ 488 and propidium iodide (PI)-negative cells by flow cytometry 24 h following irradiation with a dose of 1 Gy. (**A**) Representative flow cytometry histograms depicting the percentage of viable MOLT-4 cells are shown. (**B**) The bar graph represents the percentage of viable MOLT-4 cells detected by flow cytometry. Results are shown as the mean ± SD from four experiments. *—significantly different from irradiation alone (t-test, * *p* < 0.05).

Phosphatidylserine, a major component of the inner leaflet of the cytoplasmic membrane bilayers, is frequently used as an indicator of cell death. The externalization of this phospholipid can be detected through Annexin V, which binds to phosphatidylserine in the presence of calcium. Cell viability was quantified by Annexin V-Alexa Fluor^®^ 488/propidium iodide (PI) staining and flow cytometry analysis to determine whether the tested compounds could prevent radiation-induced cell death in MOLT-4 cells. The percentage of viable cells was 92% for non-irradiated control (0.1% DMSO vehicle) and 40% for untreated irradiated cells (1 Gy) (Figure 2A). A 60-min pre-treatment with the compounds at 100 μM significantly increased cell viability after irradiation (1 Gy), with the following values: 61% (compound **4**), 69% (compound **6**), 64% (compound **7**), 57% (compound **8**), and 51% (compound **10**) (Figure 2B). Compound **9** exhibited no significant radioprotective effect in vitro. Although compound **5** did not show statistically significant radioprotective effect (59%), it was further evaluated in vivo due to its low cytotoxicity. 

### 2.6. The Compounds Administered to Mice at MTD Caused No Pathologies

Mild to moderate signs of intoxication could be observed once the MTD (maximum tolerated dose) was reached. In vivo, MTD is described as the highest dose of a given compound that can be administered before showing signs of toxicity (based on the same dose regimen or series) and whose excess would be expected to be fatal, yielding unreliable results [18]. Therefore, a staged approach (including core and intermediate dose levels) has become a suitable strategy in this regard. 

The symptoms described in Tables S1 (in Supplementary Materials) and S2 (in Supplementary Materials) diminished spontaneously within 2 h. After observing these symptoms, MTD thresholds were set at 100, 200, 100, 200, 2000, and 650 mg/kg for compounds **4**, **5**, **6**, **7**, **8**, and **10**, respectively. Animals receiving MTD were further examined by necropsy 48 h after administration. Plasma (reflecting biochemical changes in blood), intestine with mesentery (site of administration), and liver and kidney (elimination organs) were collected to evaluate the toxic potential of the tested compounds. For evaluation of the impact of the compounds on organ function, we performed blood biochemistry test 48 h after administration. The test focused on seven important indicators: glucose and amylase for normal function of pancreas, alanine aminotransferase (ALT), aspartate aminotransferase (AST), and alkaline phosphatase (ALP) for the normal function of liver, and urea and creatinine for the normal function of kidneys. According to necropsy, blood biochemistry and histopathology analyses, the tested compounds caused no pathologies, were found harmless, and were suitable for in vivo survival tests (Table S3 in Supplementary Materials). The only histopathological change observed was limited focal necrosis in one male administered with compound **4** (one small focus), one female administered with compound **10** (five small foci), and one control male (two small foci). According to Thoolen et al. [19], focal necrosis can be occasionally seen in untreated rodents and therefore is not pathognomonic of hepatotoxicity, which is consistent with our findings.

### 2.7. Pre-Treatment with the Compounds Increased Survival of Whole-Body Irradiated Mice

To estimate the radioprotective effect on whole-body irradiated mice, compounds **4** (50 mg/kg), **5** (100 mg/kg), **6** (50 mg/kg), **7** (100 mg/kg), **8** (1000 mg/kg), and **10** (325 mg/kg) were injected (intraperitoneally, i.p.) 5 min before receiving 7.15 Gy of gamma radiation. These compounds were selected based on the cytotoxicity screening in vitro, and applied at a dose corresponding to 50% of the MTD in vivo. Notably, the tested compounds did not affect the survival of non-irradiated mice. The follow-up period was 30 days.

The lethal effect of IR could be observed after 10 days in the non-treated group, and only four mice remained alive after the 30-day period of the study. Mice pre-treated with the compounds retained longer (or equal) survival than the non-treated control with the exception of the group pre-treated with compound **7**, which showed an earlier lethal effect at 9 days post-irradiation. In addition, compounds **5** and **6** caused a decline in survival at the same time as the non-treated control (10 days post-irradiation). In contrast, the groups pre-treated with **4**, **8**, and **10** had a higher survival rate, with a drop after day 12 post-irradiation.

**Figure 3:**
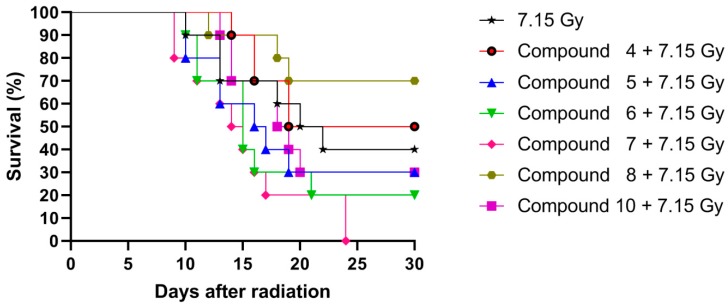
Kaplan–Meier survival curves of irradiated mice pre-treated intraperitoneally (i.p.) with saline buffer (7.15 Gy) and with compounds **4**, **5**, **6**, **7**, **8**, and **10**. Each group (n = 10) was observed until termination of the experiment (30 days after irradiation).

At the end of experiment (day 30 post-irradiation), the survival values were as follows: control (40%), **4** (50%), **5** (30%), **6** (20%), **7** (0%), **8** (70%), and **10** (30%). In summary, although compounds **4** and **10** led to a higher survival rate in the short-term, it was compound **8** that conferred a radioprotective effect and showed the highest survival values after 30 days of irradiation; however, these increases were not found to be statistically significant (Figure 3).

## 3. Discussion

In recent years, medical radiation uses have increased significantly together with the risk of radiation/nuclear events. Current pre-clinically tested radioprotectors for radiotherapy involve immune modulators, nutraceuticals (isoflavonoids), free-radical scavengers, corticosteroids, or recombinant cytokines. Several promising radioprotective agents have been synthesized de novo.

Ex-RAD(^®^), which is also known as the sodium salt of 4-carboxystyryl-4-chlorobenzylsulfone, was reported as a small molecule with radioprotective properties based on targeting p53 and its downstream regulators [20]. Ex-RAD prevented radiation-induced apoptosis and increased the survival rate of whole-body irradiated mice [21]. Tang et al. prepared a group of novel benzyl naphthyl sulfoxide derivatives from Ex-RAD. Interestingly, one of the new derivatives exhibited a remarkable increase (100%) in mice survival after irradiation [22].

Another attempt to develop radioprotective agents was made by Hosseinimehr, who synthesized a group of 2-imino-3-[(chromone-2-yl)carbonyl] thiazolidines. Some of these compounds protected mice from whole-body gamma irradiation with high statistical significance [23].

Obviously, numerous papers were published regarding novel agents for enhanced radiation protection in the last decade; notwithstanding, the only radioprotector approved by the FDA is amifostine [24].

Amifostine was reported to possess a high dose-reducing factor and to prevent radiation-induced injury repeatedly [25,26]. However, FDA has approved it for limited clinical indications only and not for non-clinical applications. Despite the efforts that have been put into increasing amifostine effectiveness, the issues with its toxicity and side effects have not been resolved. Thus, further studies are needed to improve the drug design and safety of future radioprotectors.

When searching for appropriate drug targets in radioprotection, many researchers turned their attention toward the regulation of apoptotic machinery, as both acute and chronic radiotoxicity has been associated with cell death. IR induces apoptosis through a complex DNA damage pathway. While this effect is desirable during the radiotherapy of cancer cells, it leads to radiotoxicity in normal healthy tissues. 

There has been growing evidence that a complex of pro-apoptotic and anti-apoptotic proteins (e.g., PUMA-Bcl-2, PUMA-Mcl-1) play the crucial role in apoptosis regulation, and their selective inhibition represents an exciting therapeutic opportunity [27].

Consistently, according to our previous observations, we identified PUMA together with its interaction partners as the key molecule of radiation-induced cell death signaling, and thus a promising target in medical radioprotection, but also in the therapy of neurodegenerative and cardiovascular diseases [28].

Previously, we reported a group of 1-(4-(2-hydroxyethyl)piperazin-1-yl)-3-phenoxypropan-2-ol derivatives with a possible mechanism of radioprotective effect based on interference with Bcl-2 family protein–protein interaction [14]. The goal of this work was to describe the second generation of piperazine derivates, as we anticipate a similar mechanism of action with an improved pharmacological profile. Although the molecular explanation of the potential radioprotective effect is a subject of parallel study (currently undergoing), the molecular docking data indicate possible interaction with the hydrophilic pocket of anti-apoptotic protein Bcl-2.

In the presented study, 10 new compounds were synthesized based on 1-(2-hydroxyethyl)piperazine derivatives. Compound **2** has already been published as a cholinesterase inhibitor with intended application for the treatment of Alzheimer’s disease [15]. Since it contained a piperazine structural moiety linked through an aminoethyl group to an aromatic moiety, we presumed its inhibition of other protein–protein interactions. Unfortunately, the compound was discarded in baseline screening due to high cytotoxicity.

Therefore, the substance was modified by substituting the piperazine core with a hydroxyethyl group. By substituting the secondary amino group of the piperazine of compound **2**, we presumed that there would be a reduction in the cytotoxic effect. This phenomenon was confirmed during the baseline screening but at a higher concentration (100 µM). Compound **3** was eventually found to be excessively toxic to the cell lines and excluded from further testing. 

Compound **9** showed no radioprotective effect in vitro. A possible explanation could be that the compound does not contain a basic piperidine moiety, which seems to be essential for this property. Such phenomenon has already been published by our group [14]. Compound **9** could not be tested in vivo due to the low solubility. Compared to the other compounds, the low solubility was due to the presence of two aromatic moieties that also significantly affect the ClogP value (Table 1). All other substances (**4**, **5**, **6**, **7**, **8**, and **10**) have been tested in vitro and in vivo based on cytotoxic screening and physicochemical properties.

For structurally similar substances **5**, **6**, **7**, and **10**, there is an evident correlation between ClogP and cytotoxic effect on various cell lines. Compound **6**, substituted with a naphthalene moiety (with the highest ClogP and hence MTC), appears to be the most promising radioprotector of all the compounds evaluated in vitro. On the other hand, no significant effect was observed under in vivo conditions. The effect is comparable to substances **5** and **7**. According to the results for compounds **5** and **10**, the presence or absence of a hydroxyl group on the linker plays no significant role concerning the potential radioprotective effect.

Substance **4** is structurally comparable to those prepared recently by our group [14]. In addition, a halogen atom (Cl) was added to the structure, which could influence other binding interactions. In vitro and in vivo tests indicate that the compound (despite its relatively higher toxicity) possesses a radioprotective effect in vivo.

The structurally different compound **8** contains no aromatic moiety. This compound was synthesized by linking two 1-(2-hydroxyethyl)piperazine moieties with an alkyl linker. The compound is minimally non-toxic and excellently soluble in media. After in vivo evaluation, it appeared to be the most effective compound in this group in terms of radioprotection. In addition, according to the safety profile, it might be considered an appropriate compound for further studies. 

Regarding the structure–activity relationship, we observed that acridine moiety increased the cytotoxicity of the compounds **2** and **3** and therefore were omitted. Interestingly, lower cytotoxicity was achieved by substitution of the secondary amine at the 4-position of piperazine nitrogen, whereby a tertiary amine was formed (compound **3**). The radioprotective properties are reduced in compounds with the methoxy group. Furthermore, the 1-(2-hydroxyethyl)piperazine moiety seems to be crucial, as the compounds lacking this structure possess increased cytotoxicity and insolubility (compound **9**). The longest survival was found after the treatment with compound **8.** Moreover, it is lacking aromatic moieties and is entirely non-toxic and well soluble. Thus, it might be the most promising radioprotective compound that is suitable for further development. 

## 4. Experimental Section

### 4.1. Synthesis and Analysis

Analytical grade reagents were purchased from Sigma-Aldrich, Fluka and Merck (Darmstadt, Germany). The solvents were purchased from Penta Chemicals (Prague, Czech Republic). Reactions were monitored by thin layer chromatography (TLC) using a precoated silica gel 60 F254 TLC aluminium sheet. Column chromatography was performed with silica gel 0.063–0.200 mm. Melting points were determined on a microheating stage PHMK 05 (VEB Kombinat Nagema, Radebeul, Germany) and are uncorrected. All compounds were fully characterized by NMR spectra and HRMS. NMR spectra were recorded on a Varian VNMR S500 (operating at 500 MHz for ^1^H and 126 MHz for ^13^C; Varian Comp., Palo Alto, CA, USA). The chemical shifts (*δ*) are given in ppm and related to tetramethylsilane (TMS) as an internal standard. Coupling constants (*J*) are reported in Hz. Splitting patterns are designated as s, singlet; d, doublet; t, triplet; dd, doublet of doublets; and m, multiplet. Mass spectra were recorded using a combination of liquid chromatography and mass spectrometry: high-resolution mass spectra (HRMS) and sample purities were obtained by high-performance liquid chromatography (HPLC) with UV and the mass spectrometry (MS) gradient method. The system used in this study was a Dionex Ultimate 3000 UHPLC: RS Pump, RS Column Compartment, RS Autosampler, Diode Array Detector, Chromeleon software (version 6.80 SR13 build 3967, Thermo Fisher Scientific, Germering, Germany) with a Q Exactive Plus Orbitrap mass spectrometer with Thermo Xcalibur software (version 3.1.66.10, Thermo Fisher Scientific, Bremen, Germany). Detection was performed by mass spectrometry in positive mode. Settings of the heated electrospray source were: spray voltage 3.5 kV, capillary temperature 220 °C, sheath gas 55 arbitrary units, auxiliary gas 15 arbitrary units, spare gas 3 arbitrary units, probe heater temperature 220 °C, max spray current 100 µA, S-lens RF Level 50. A C18 column (Kinetex EVO C18, 3 × 150 mm, 2.6 µm, Phenomenex, Japan) was used in this study. Mobile phase A was ultrapure water of ASTM I type (resistance 18.2 MΩ.cm at 25 °C) prepared by a Barnstead Smart2Pure 3 UV/UF apparatus (Thermo Fisher Scientific, Bremen, Germany) with 0.1% (*v*/*v*) formic acid (LC-MS grade, Sigma Aldrich, Steinheim, Germany); mobile phase B was acetonitrile (MS grade, Honeywell-Sigma Aldrich, Steinheim, Germany) with 0.1% (*v*/*v*) of formic acid. The flow was constant at 0.4 mL/min and began with 1 min of isocratic flow of 10% B, and then 3 min of gradient to 100% B, followed by 1 min of constant 100% B. Then, the composition reverted to 10% B and was equilibrated for 2.5 min. Samples were dissolved in methanol (LC-MS grade, Fluka-Sigma Aldrich, Steinheim, Germany) at a concentration of 1 mg/mL, and sample injection was 1 µl. Purity was determined from UV spectra measured at a wavelength of 254 nm. HRMS was determined by the total ion current spectra from the mass spectrometer. Clog *P* was calculated with MarvinSketch software (version 14.9.8.0, ChemAxon, Budapest, Hungary).

7-Methoxy-*N*-(2-(piperazin-1-yl)ethyl)-1,2,3,4-tetrahydroacridin-9-amine trihydrochloride (**2**). The synthetic procedure has already been published elsewhere [15]. The product was obtained as a yellow solid (0,49 g, 78%), mp = 172–173 °C; ^1^H-NMR (methanol-*d*_4_): δ 7.72 (d, 1H, *J* = 9.2 Hz), 7.55 (d, 1H, *J* = 2.7 Hz), 7.40 (dd, 1H, *J* = 9.2, 2.7 Hz), 3.96 (s, 3H), 3.86 (m, 2H), 3.78 (m, 2H), 2.98 (m, 6H), 2.78 (m, 2H), 2.59 (m, 4H), 1.96 (m, 4H); ^13^C-NMR (126 MHz, cd_3_od): δ 158.1, 154.9, 151.6, 132.8, 125.6, 123.7, 120.4, 115.7, 104.2, 58.7, 52.9, 45.9, 45.2, 41.4, 31.5, 25.5, 22.8, 22.1; HRMS: *m/z* 341.2330 [M + H]^+^ (calculated for [C_20_H_29_N_4_O]^+^ 341.2336).

7-Methoxy-*N*-{2-[4-(2-hydroxyethyl)piperazin-1-yl]ethyl}-1,2,3,4-tetrahydroacridin-9-amine trihydrochloride (**3**). Hunig’s base (0.45 g, 3.6 mmol) and 2-bromoethanol (1.8 mmol) were added to intermediate **2** (0.2 g, 0.6 mmol) dissolved in anhydrous CH_2_Cl_2_. The reaction mixture was refluxed under nitrogen overnight, followed by the evaporation of volatile solvents under reduced pressure. The crude product was purified by column chromatography eluting with EtOAc/MeOH/NH_3_ (25% aq.) (40:1:0.2→15:1:0.2) to obtain a yellow oily residue. The resulting free base was dissolved in methanol and treated with hydrochloric acid to give **3** as a yellow solid (0.17 g, 75%), m.p. = 157–158 °C; ^1^H-NMR (500 MHz, methanol-*d*_4_) δ 7.79 (d, 1H, *J* = 9.2 Hz), 7.76 (d, 1H, *J* = 2.7 Hz), 7.54 (dd, 1H, *J* = 9.2, 2.7 Hz), 4.35 (t, *J* = 6.2 Hz, 2H), 4.04 (s, 3H), 3.98–3.93 (m, 2H), 3.47–3.41 (m, 2H), 3.09 (t, *J* = 6.0 Hz, 2H), 2.91 (t, *J* = 5.7 Hz, 2H), 1.98 (m, 12H), 1.43–1.36 (m, 2H); ^13^C-NMR (126 MHz, methanol-*d*_4_): δ 158.2, 154.7, 152.6, 133.1, 125.5, 123.7, 120.4, 115.7, 104.2, 61.5, 58.7, 52.9, 45.9, 45.2, 41.4, 35.2 31.5, 25.5, 22.6, 22.2; HRMS: *m/z* 385.2590 [M + H]^+^ (calculated for [C_22_H_33_N_4_O_2_]^+^ 385.2598).

1-(4-chloro-3-methylphenoxy)-3-(4-(2-hydroxyethyl)piperazin-1-yl)propan-2-ol dihydrochloride (**4**). 1-(2-hydroxyethyl)piperazine **1** (0.2 g, 1.5 mmol) was dissolved in anhydrous acetonitrile and K_2_CO_3_ (0,4 g. 3.0 mmol) with appropriate intermediate **4b** (0.2g, 1 mmol) was added. The reaction mixture was stirred under nitrogen for 4 h. The mixture was filtered and the filtrate was concentrated under reduced pressure. The residue was purified by column chromatography eluting with EtOAc/MeOH/NH_3_ (25% aq) (6:2:0.2) to obtain a colorless oily residue. The resulting free base was dissolved in methanol and treated with hydrochloric acid to give **4** as white solid. (0.27 g, 79%) m.p. = 189–190 °C; ^1^H-NMR (500 MHz, methanol-*d*_4_) δ 7.23 (d, *J* = 8.7 Hz, 1H), 6.90 (d, *J* = 3.0 Hz, 1H), 6.78 (dd, *J* = 8.8, 3.0 Hz, 1H), 4.20–4.12 (m, 1H), 4.02–3.92 (m, 2H), 3.82–3.79 (m, 2H), 3.14–3.00 (m, 6H), 2.96–2.89 (m, 6H), 2.82–2.70 (m, 2H), 2.33 (s, 3H); ^13^C-NMR (126 MHz, methanol-*d*_4_) δ 158.97, 138.08, 130.63, 126.93, 118.17, 114.53, 71.82, 67.85, 61.03, 60.09, 57.91, 53.23, 52.65, 20.28; HRMS: *m/z* 329.1620 [M + H]^+^ (calculated for [C_16_H_26_ClN_2_O_3_]^+^ 329.1621).

General procedure for the synthesis of 2-(4-(3-(arylamino)propyl)piperazin-1-yl)ethanol trihydrochloride derivatives (**5**–**7**). 1-Bromo-3-chloropropane (1 mmol) was dissolved in MeCN (anhydrous, 5 mL) and KI (potassium iodide, 0.1 mmol) was added. After 5 min of stirring, the solution of appropriate arylamine (aniline **5a**, naphtyl-1-amine **6a** or *p*-anisidine **7a**) (3 mmol) in MeCN (anhydrous, 10 mL) was added. The final mixture was stirred under microwave conditions (110 °C, 15 min). The mixture was filtered and the filtrate was concentrated under reduced pressure. The residue was purified by column chromatography eluting with petroleum ether/EtOAc (20:1) to obtain intermediates **5b**, **6b**, or **7b** as a yellow (**6b** violet) oily residue in a yield around 30%.

1-(2-hydroxyethyl)piperazine **1** (1.5 mmol) was dissolved in anhydrous MeCN (20 mL) and K_2_CO_3_ (3.0 mmol) and the appropriate intermediate (**5b**, **6b** or **7b**) were added. The reaction mixture was stirred under nitrogen for 4 h. The mixture was filtered, and the filtrate was concentrated under reduced pressure. The residue was purified by flash chromatography eluting with petroleum ether/EtOAc (10:1) to obtain yellow or white residues in 61–69% yields. The resulting free bases were dissolved in methanol and treated with hydrochloric acid to give **5–7** as yellow or white solids.

2-(4-(3-(phenylamino)propyl)piperazin-1-yl)ethanol trihydrochloride (**5**). White solid (0.18 g, 68%) m.p. = 185–186 °C; ^1^H-NMR (500 MHz, chloroform-*d*) δ 7.21–7.16 (m, 2H), 6.73–6.67 (m, 1H), 6.64–6.59 (m, 2H), 3.66–3.62 (m, 2H), 3.20 (t, *J* = 6.4 Hz, 2H), 2.61–2.57 (m, 2H), 2.69–2.36 (m, 8H), 2.51 (t, *J* = 6.7 Hz, 2H), 1.82 (p, *J* = 6.5 Hz, 2H); ^13^C-NMR (126 MHz, chloroform-*d*) δ 148.7, 129.2, 117.0, 112.6, 59.2, 57.7, 57.2, 53.2, 52.9, 43.4, 25.8; HRMS: *m/z* 264.2065 [M + H]^+^ (calculated for [C_15_H_26_N_3_O]^+^ 264.2070).

2-(4-(3-(naphthalen-1-ylamino)propyl)piperazin-1-yl)ethanol trihydrochloride (**6**). White solid (0.22 g, 69%) m.p. = 181–182 °C; ^1^H-NMR (500 MHz, chloroform-*d*) δ 8.00–7.96 (m, 1H), 7.79 (dd, *J* = 7.9, 1.5 Hz, 1H), 7.48–7.38 (m, 2H), 7.38–7.33 (m, 1H), 7.21 (d, *J* = 8.1 Hz, 1H), 6.56 (d, *J* = 7.5 Hz, 1H), 3.68 (t, *J* = 5.4 Hz, 2H), 3.41–3.35 (m, 2H), 2.83–2.50 (m, 12H), 2.00 (p, *J* = 6.1 Hz, 2H); ^13^C-NMR (126 MHz, chloroform-*d*) δ 144.5, 134.5, 128.8, 127.0, 125.8, 124.4, 123.7, 121.1, 116.8, 103.8, 59.7, 58.5, 57.9, 53.6, 53.0, 44.8, 24.9; HRMS: *m/z* 314.2283 [M + H]^+^ (calculated for [C_19_H_28_N_3_O]^+^ 314.2227).

2-(4-(3-((4-methoxyphenyl)amino)propyl)piperazin-1-yl)ethanol trihydrochloride (**7**). Yellow solid (0.18 g, 61%) m.p. = 182–183 °C; ^1^H-NMR (500 MHz, chloroform-*d*) δ 6.89–6.85 (m, 2H), 6.85–6.81 (m, 2H), 3.85 (s, 3H), 3.71–3.67 (m, 2H), 3.15 (t, *J* = 6.4 Hz, 2H), 2.61–2.55 (m, 2H), 2.692.16 (m, 8H), 2.52 (t, *J* = 6.7 Hz, 2H), 1.88 (p, *J* = 6.5 Hz, 2H); ^13^C-NMR (126 MHz, chloroform-*d*) δ 149.1, 130.2, 118.0, 112.4, 59.3, 57.8, 57.2, 53.2, 52.9, 49.2, 43.5, 23.9; HRMS: *m/z* 294.2172 [M + H]^+^ (calculated for [C_16_H_28_N_3_O_2_]^+^ 294.2176).

1.3-bis [4-(2-hydroxyethyl)piperazin-1-yl)propan-2-ol tetrahydrochloride (**8**). 1-(2-hydroxyethyl)piperazine **1** (1 mmol) dissolved in anhydrous MeOH (0.9 mL) was treated with 1,3-dibromo-2-propanol (1 mmol) at room temperature and the mixture was stirred for 48 h. Silica gel was added to the mixture and the solvent was evaporated. The residue was purified by column chromatography using CH_2_Cl_2_/MeOH/NH_3_ (25% aq.) (5:1:0.2) as the eluent to obtain colorless oily residue. The resulting free base was dissolved in methanol and treated with hydrochloric acid to give **8** as a colorless oil. (85 mg, 27%) ^1^H-NMR (500MHz; DMSO-*d*_6_) δ 3.73 (m; 1H), 3.47 (t; 4H; *J* = 6.3 Hz), 2.41 (m; 16H), 2.36 (t; 4H; *J* = 6.3 Hz), 2.30 (m; 2H), 2.20 (m; 2H); ^13^C-NMR (126 MHz; DMSO-*d*_6_) δ 65.1, 62.8, 60.2, 58.4, 53.3, 53.1; HRMS: *m/z* 317.2543 [M + H]^+^ (calculated for [C_15_H_33_N_4_O_3_]^+^ 317.2542).

1,3-bis(phenylamino)propan-2-ol dihydrochloride (**9**). Epibromohydrin (1 mmol) was slowly added with stirring to aniline (1 mmol) at 0 °C, and the final mixture was stirred for 1 h at 50 °C using a microwave reactor. Then, the reaction mixture was diluted with MeOH, silica gel was added, and the mixture was adsorbed onto silica gel using a rotary vacuum evaporator. The product was purified by column chromatography using CH_2_Cl_2_/MeOH/NH_3_ (25% aq.) (300:1:0.1) as eluent to obtain a colorless oily residue. The resulting free base was dissolved in methanol and treated with hydrochloric acid to give **9** as a colorless oil. (61 mg, 25%); ^1^H-NMR (500MHz; DMSO-*d*_6_) δ 7.05 (m; 4H), 6.59 (m; 4H), 6.51 (m; 2H), 3.83 (m; 1H), 3.49 (m; 2H), 3.16 (m; 2H); ^13^C-NMR (126 MHz; DMSO-*d*_6_) δ 148.4, 128.8, 115.9, 112.2, 68.3, 47.2; HRMS: *m/z* 243.1488 [M + H]^+^ (calculated for [C_15_H_19_N_2_O]^+^ 243.1492).

1-(phenylamino]-3-[4-(2-hydroxyethyl)piperazin-1-yl)propan-2-ol trihydrochloride (**10**). Epibromohydrin (1 mmol) was slowly added with stirring to aniline **5a** (1 mmol) at 0 °C, and the final mixture was stirred for 1 h at 50 °C using a microwave reactor. Then, the reaction mixture was diluted with MeOH, silica gel was added, and the mixture was adsorbed on to silica gel using a rotary vacuum evaporator. The product was purified by column chromatography using CH_2_Cl_2_/MeOH/NH_3_ (25% aq.) (300:1:0.1) as eluent to obtain intermediate **10b** as a colorless oily residue in a yield of 45%. The intermediate (1-Bromo-3-(phenylamino)propan-2-ol **10b** (1 mmol) was dissolved in anhydrous MeCN (4.5 mL) and K_2_CO_3_ (1.5 mmol) was added at 0 °C and stirred for 15 min. Then, a solution of 1-(2-hydroxyethyl)piperazine **1** (2 mmol) in anhydrous MeCN (2.5 mL) was added dropwise at 0 °C, and the final mixture was stirred overnight from 0 °C to room temperature. The solid residue was removed by filtration under reduced pressure, and the filtrate was evaporated. The mixture was purified by column chromatography using CH_2_Cl_2_/MeOH/NH_3_ (25% aq.) (5:1:0.2) as eluent to yield 251 mg of product as a colorless oil. The resulting free base was dissolved in methanol and treated with hydrochloric acid to give **10** as a white solid. (0.25 g, 90%) m.p. = 154–155 °C; ^1^H-NMR (500MHz; DMSO-*d*_6_) δ 7.05 (m; 2H), 6.58 (m; 2H), 6.51 (m; 1H), 5.44 (m; 1H), 3.48 (m; 2H), 3.11 (m; 1H), 2.92 (m; 1H), 2.40 (m; 8H), 2.35 (m; 2H), 2.29 (m; 2H); ^13^C-NMR (126 MHz; DMSO-*d*_6_) δ 149.9, 128.8, 115.5, 112.0, 66.2, 62.6, 60.3, 58.5, 53.5, 53.3, 48.6; HRMS: *m/z* 280.2016 [M + H]^+^ (calculated for [C_15_H_26_N_3_O_2_]^+^ 280.2008).

### 4.2. Molecular Docking

The structure of receptor was gained from the Protein Data Bank, PDB ID 4LXD (Bcl_2-Navitoclax Analog complex) [29]. The structure has a resolution of 1.9 Å and it does not have any Ramachandran’s outliers. Therefore, it was found to be suitable for molecular docking. The structure was prepared by the DockPrep function of UCSF Chimera (version 1.4) and converted to pdbqt-files by AutodockTools (v. 1.5.6) [30,31]. Three-dimensional structures of ligands were minimized by Avogadro (v. 1.1.0) and converted to a pdbqt-file format by Open Babel (v. 2.3.1) [32,33]. The docking calculations were done by Autodock Vina (v. 1.1.2) with the exhaustiveness of 8 [34]. Calculation was repeated 10 times for each ligand, and the best-scored result was selected for manual inspection. The visualization of enzyme–ligand interactions was prepared using Pymol (v. 1.7.4.5) [The PyMOL Molecular Graphics System, Version 1.7.4.5, Schrödinger, LLC, Mannheim, Germany].

### 4.3. Cell Culture and Treatment with Novel Compounds

The cell lines Jurkat, MOLT-4, A2780, A549, HT-29, PANC-1, HeLa, MCF-7, SAOS-2, and MRC-5 (all purchased from Sigma Aldrich, Czech Republic) were included in this study. All cell lines were maintained according to the provider’s guidelines, and plated and treated in 96-well plates (500 to 30 × 10^3^ cells per well). The tested compounds **2**, **3**, **4**, **5**, **6**, **7**, **8**, **9**, and **10** were dissolved in 0.1% DMSO and kept in 10 mM stock solutions. Before use, the stock solution was diluted in culture media at ratios of 1:100 and 1:1000. The studied cell lines were treated with the compounds **2, 3, 4, 5, 6, 7, 8, 9**, and **10** at concentrations of 10 and 100 µM for 48 h. Control cells were tested in culture media with and without DMSO (0.1%). The cells were also treated with 1 µM doxorubicin as a cytotoxicity control. 

### 4.4. Cytotoxicity of Novel Compounds In Vitro

The WST-1 (2-(4-iodophenyl)-3-(4-nitrophenyl)-5-(2,4-disulfophenyl)-2H-tetrazolium, monosodium salt) test (Roche, Mannheim, Germany) was used according to the manufacturer’s protocol to determine cell viability in all 10 cell lines 48 h after treatment with the tested compounds. The cells were analyzed in a Tecan Infinite M200 spectrometer (Tecan Group, Männedorf, Switzerland) at 440 nm.

IC_50_ and MTC were determined through MTT assays. The MTT assay was performed according to [35]. In brief, the A549 cell line was plated in 96-well plates (100 µL, 9 × 10^3^ cells per well) and incubated overnight. The tested compounds were prepared in phosphate buffer saline (PBS; Sigma–Aldrich) and serially diluted in DMEM (Dulbecco’s Modified Eagle Medium). An untreated control was included as a viability standard in each plate. After 24 h of treatment, the viability of A549 cells was measured at 570 nm. All experiments were performed in triplicate. The GraphPad Prism statistics software v5.04 (GraphPad Software Inc., San Diego, CA, USA) was used to calculate the IC_50_ values for parameter nonlinear regression. Values are expressed as mean ± SEM.

### 4.5. Evaluation of Radioprotection In Vitro

Fresh 50 mM stock solutions of the compounds were prepared in DMSO (Sigma-Aldrich, St. Louis, MO, USA) before use in the study. For the radioprotection experiments, MOLT-4 cells were treated with 100 μM solutions of the novel compounds and irradiated after 60 min with a ^60^Co γ-ray source (Chisotron Chirana, Prague, Czech Republic). The non-irradiated and irradiated control cells were treated with DMSO vehicle only (0.1%) and handled in parallel with the experimental cells. Non-irradiated control cells were handled in the same manner. Cell viability was determined by flow cytometry using an Alexa Fluor^®^ 488 Annexin V/Dead Cell Apoptosis kit (Life Technologies, Grand Island, NY, USA) according to the manufacturer’s instructions. The Alexa Fluor^®^ 488 Annexin V/Dead Cell Apoptosis kit employs the property of Alexa Fluor^®^ 488 conjugated to Annexin V to bind to phosphatidylserine in the presence of Ca^2+^, and the property of propidium iodide (PI) to enter cells with damaged cell membranes and bind to DNA. Measurement was performed using a CyAn (Beckman Coulter, Miami, FL, USA) flow cytometer. Listmode data was analyzed with the Kaluza Analysis v1.3 software (Beckman Coulter, Miami, FL, USA).

### 4.6. Safe Use Evaluation In Vivo

Both male and female BALB/c mice (Velaz s r.o., Prague, Czech Republic) with an average weight of 21 ± 0.8 g and 21 ± 0.8 g, respectively, were selected for the safe use evaluation in vivo. The mice were housed in environmentally controlled breeding units (Faculty of Military Health Sciences vivarium; temperature 21 ± 1 °C, 12/12 h light/dark cycle) with access to food (Cerea corp., Pardubice, Czech Republic) and water ad libitum. The acclimatization period of the mice was at least 10 days before each experiment. All experimental procedures and protocols were reviewed and approved by the Ethics Committee of the Faculty of Military Health Sciences in Hradec Kralove, University of Defence in Brno. Compounds **2**, **3**, **4**, **5**, **6**, **7**, **8**, **9**, and **10** were dissolved in physiological saline solution (B. Braun Melsungen AG, Melsungen, Germany) and injected intraperitoneally (i.p.) into the corresponding mouse groups, which were composed of two males and two females. Several doses were administered to determine the maximum tolerated dose (MTD), which was assessed by close observation for signs of toxicity within the first two hours after administration of the compounds, and then regularly over the following 48 h. The observed symptoms were registered according to the laboratory Animal Science Association UK guidelines (for a detailed description, see [36]. After 48 h, the surviving animals were euthanized with CO_2_ and evaluated by basic macroscopic necropsy. Venous blood was collected into heparinized tubes (Scanlab Systems, Prague, Czech Republic) and the plasma was separated in a U-320R centrifuge (Boeco, Hamburg, Germany) to further evaluate the toxic potential of the tested compounds. The concentrations of glucose, urea, and creatinine and the levels of alanine aminotransferase (ALT), aspartate aminotransferase (AST), alkaline phosphatase (ALP), and amylase were evaluated in a qualified biochemical laboratory of the University Hospital Hradec Kralove (Hradec Kralove, Czech Republic). Additionally, intestine, liver, and kidney samples were collected for histopathologic examination. The samples were fixed in 10% neutral buffered formalin, dehydrated through increasing concentrations of ethanol and xylene, and transferred into liquid paraffin (all from Bamed, Ceske Budejovice, Czech Republic) using a Leica TP1020 tissue processor (Leica, Wetzlar, Germany). The paraffin-embedded samples were cut into 5 µm thick sections (Microtome model SM2000 R, Leica, Wetzlar, Germany), re-hydrated (xylene and decreasing ethanol concentrations), stained with hematoxylin and eosin (both from Merck, Darmstadt, Germany), dehydrated once again, and mounted in DPX (Dibutylphthalate Polystyrene Xylene) media (Merck). The histopathological analysis was performed on a BX-51 microscope (Olympus, Tokyo, Japan).

### 4.7. Animals and Gamma Radiation

Adult female BALB/c mice (21 ± 0.8 g) were used for all experiments (Velaz a.s., Prague, Czech Rep.). All the performed methods were in accordance with NIRS Guidelines for the Care and Use of Laboratory Animals and approved by the Committee for animal rights of the Ministry of Defence of the Czech Republic no. ČJ MO 255172/2019-684800. The mice, arranged in groups of 10 animals each, received a dose of 7.15 Gy from a ^60^Co gamma source (Chisotron, Chirana, Czech Republic) at a dose rate of 1.04 Gy/min. Non-irradiated and non-treated control groups were included. Their survival and health status were checked on a daily basis for 30 days.

### 4.8. Preparation of the Novel Compound Solutions and Evaluation of Radioprotection In Vivo

Compounds **4** (50 mg/kg), **5** (100 mg/kg), **6** (50 mg/kg), **7** (100 mg/kg), **8** (1000 mg/kg), and **10** (325 mg/kg) were selected and administered i.p. 5 min before irradiation. The doses were prepared from 100 mg/mL stock before each experiment.

### 4.9. Statistical Analysis

The charts were made with the GraphPad Prism 6 biostatistics software (GraphPad Software, La Jolla, CA, USA). The evaluated groups were analyzed by Student’s t-test or one-way ANOVA, followed by a post hoc Tukey test (*p* < 0.05).

## 5. Conclusions 

In summary, all the compounds were tested in vitro, revealing that compounds **2** (10 µM) and **3** (100 µM) were highly cytotoxic, while compound **9** was not cytotoxic, but was insufficiently soluble for necessary concentrations, making it inappropriate for further tests in vivo. After determining the MTD in vivo of the remaining compounds (**4**, **5**, **6**, **7**, **8**, and **10**), we used only 50% of the calculated MTD and assessed the radioprotective effect through the long-term survival of the irradiated mice.

Our results show that compounds **4** and **10** were able to prolong survival but failed to prevent fatal radiation-induced injury in the long term. On the other hand, pre-treatment with compound **8** led to an increased survival rate in the irradiated animals. Although this increase was not statistically significant, we propose compound **8** as a valuable candidate for further research due to its low toxicity and appropriate yield during synthesis.

## Figures and Tables

**Table 1:** Structures, yields, and ClogP of prepared compounds.

Compound	Yield (%)	Melting Point (°C)	*ClogP
**2**	78	172–173	2.255
**3**	75	157–158	1.948
**4**	79	189–190	1.478
**5**	68	185–186	0.682
**6**	69	181–182	1.671
**7**	61	182–183	0.524
**8**	27	oil	−2.107
**9**	25	oil	2.090
**10**	90	154–155	−0.009

* ClogP was calculated with MarvinSketch (version 14.9.8.0) software.

**Table 2:** Cytotoxic effect of novel compounds in vitro. Values represent cell viability after treatment with 10 µM (**A**) and 100 µM (**B**) of the tested compounds. The results are expressed as relative viability (%) when compared to an untreated control (100%). Compound **2** was not tested at 100 µM. DOX—doxorubicin (1 µM). Each value represents the mean of three independent experiments ± SD. Values from the intervals 0–25% (red color), 26–50% (orange color) and 50–75% (yellow color) are highlighted with different colors.

(**A**)										
**Cell line**	**2**	**3**	**4**	**5**	**6**	**7**	**8**	**9**	**10**	**DOX**
Jurkat	31 ± 14	95 ± 9	105 ± 5	98 ± 4	101 ± 1	100 ± 5	103 ± 5	104 ± 5	108 ± 7	2 ± 0
MOLT-4	0 ± 0	81 ± 13	88 ± 6	93 ± 9	90 ± 7	93 ± 3	106 ± 8	103 ± 8	112 ± 6	1 ± 0
A549	27 ± 3	94 ± 15	100 ± 4	86 ± 7	103 ± 7	95 ± 5	97 ± 5	107 ± 11	105 ± 9	42 ± 11
HT-29	28 ± 6	85 ± 11	100 ± 2	91 ± 2	86 ± 4	96 ± 3	101 ± 1	102 ± 5	104 ± 11	55 ± 18
PANC-1	39 ± 2	78 ± 6	97 ± 7	86 ± 16	92 ± 11	99 ± 10	98 ± 9	93 ± 12	95 ± 7	78 ± 7
A2780	44 ± 9	84 ± 7	98 ± 2	93 ± 5	74 ± 3	94 ± 3	104 ± 4	90 ± 3	103 ± 6	8 ± 4
HeLa	25 ± 1	104 ± 13	106 ± 2	100 ± 4	108 ± 6	96 ± 8	103 ± 4	99 ± 9	110 ± 7	76 ± 12
MCF-7	15 ± 1	84 ± 9	105 ± 4	101 ± 6	102 ± 8	105 ± 6	101 ± 5	100 ± 4	98 ± 2	32 ± 3
SAOS-2	28 ± 2	80 ± 4	99 ± 6	96 ± 4	94 ± 6	102 ± 7	102 ± 6	106 ± 2	97 ± 6	22 ± 4
MRC-5	23 ± 2	84 ± 2	97 ± 8	98 ± 7	91 ± 4	100 ± 3	95 ± 7	102 ± 8	102 ± 3	22 ± 2
(**B**)										
**Cell line**	**2 - not tested**	**3**	**4**	**5**	**6**	**7**	**8**	**9**	**10**	**DOX**
Jurkat	x	12 ± 5	89 ± 1	98 ± 3	93 ± 2	96 ± 0	96 ± 13	79 ± 11	95 ± 4	2 ± 0
MOLT-4	x	2 ± 0	92 ± 6	104 ± 7	112 ± 4	99 ± 3	81 ± 6	71 ± 4	100 ± 4	1 ± 0
A549	x	13 ± 2	99 ± 0	104 ± 8	93 ± 3	115 ± 0	111 ± 11	98 ± 13	105 ± 2	42 ± 11
HT-29	x	14 ± 5	83 ± 4	109 ± 2	97 ± 4	118 ± 2	154 ± 11	148 ± 2	113 ± 2	55 ± 18
PANC-1	x	30 ± 4	111 ± 24	115 ± 7	111 ± 5	116 ± 3	117 ± 11	118 ± 5	111 ± 6	78 ± 7
A2780	x	20 ± 5	89 ± 1	105 ± 8	95 ± 4	91 ± 2	76 ± 10	64 ± 5	96 ± 2	8 ± 4
HeLa	x	11 ± 3	137 ± 5	116 ± 10	104 ± 7	132 ± 3	100 ± 18	90 ± 14	121 ± 3	76 ± 12
MCF-7	x	10 ± 2	90 ± 3	106 ± 1	94 ± 9	104 ± 6	96 ± 7	98 ± 11	96 ± 6	32 ± 3
SAOS-2	x	10 ± 2	88 ± 4	111 ± 6	111 ± 4	109 ± 2	87 ± 7	90 ± 4	102 ± 2	22 ± 4
MRC-5	x	9 ± 6	93 ± 5	102 ± 8	94 ± 2	104 ± 3	119 ± 16	120 ± 16	102 ± 8	22 ± 2

**Table 3:** Inhibitory concentration (IC_50_) and maximum tolerated concentration (MTC) in A549 cells.

Compounds	IC_50_ ± SEM (mM)	MTC (mM)
**2**	0.04 ± 0.002	0.002
**3**	0.14 ± 0.01	0.01
**4**	0.64 ± 0.03	0.05
**5**	1.35 ± 0.12	0.05
**6**	0.45 ± 0.01	0.02
**7**	2.55 ± 0.21	0.39
**8**	> 25	6.25
**9**	0.24 ± 0.01	0.09
**10**	5.06 ± 0.23	1.56

## Supplementary Materials

The following are available online at https://www.mdpi.com/1420-3049/25/3/532/s1, Schemes S1–S6: Synthetic pathway of novel compounds, Table S1: Symptoms induced by MTD of novel compounds in male mice, Table S2: Symptoms induced by MTD of novel compounds in female mice, Table S3: Biochemical parameters evaluated 48 h after administering MTD of novel compounds in male (m) and female (f) BALB/c mice.
