# Supplementary material for: Substituted Piperazines as Novel Potential Radioprotective Agents
Source: Molecules. 2020 Jan 25;25(3):532. doi: 10.3390/molecules25030532 (PMC7038073; doi:10.3390/molecules25030532)
Supplement: Supplementary file 1 [file molecules-25-00532-s001.zip › Supplementary data_Table_S1-S3.docx]

| signs and symptoms | Tested compound | | | | | | | | | | | |
| --- | --- | --- | --- | --- | --- | --- | --- | --- | --- | --- | --- | --- |
|  | **4**  **(100 mg/kg)** | | **5**  **(200 mg/kg)** | | **6**  **(100 mg/kg)** | | **7**  **(200 mg/kg)** | | **8**  **(2000 mg/kg)** | | **10**  **(650 mg/kg)** | |
|  | 1 | 2 | 1 | 2 | 1 | 2 | 1 | 2 | 1 | 2 | 1 | 2 |
| hypersalivation |  |  |  |  |  |  |  |  |  |  |  |  |
| hyper/hypoventilation | + | + |  | ++ | ++ | ++ | ++ | ++ |  |  | + | + |
| exopthalmus |  |  |  |  |  |  |  |  |  |  |  |  |
| drop of eyelids |  |  | + | + | + |  |  | + |  |  |  |  |
| lacrimation |  |  | + |  |  |  |  |  |  |  |  |  |
| piloerection |  |  |  |  |  | + |  |  |  |  |  |  |
| acrocyanosis |  |  |  |  |  |  |  |  |  |  |  |  |
| ataxia |  |  | ++ | + | + | + |  |  |  |  | + | + |
| prostration | + | + |  | ++ | + | + | ++ | ++ |  |  | ++ | ++ |
| tremor |  |  | ++ | ++ |  | + | ++ | ++ | + | + | + | + |
| convulsion |  |  |  |  |  |  |  |  |  |  |  |  |
| excitation |  |  |  |  |  |  |  |  | + | + | + | + |
| saber-like tail |  |  | + |  |  |  |  |  |  |  |  |  |
| stooping |  |  |  |  | ++ |  |  |  |  |  |  |  |
| weight at 24 h (%) | -2.6 | -3.0 | +2.3 | -1.2 | +1.0 | -2.7 | -0.3 | -1.5 | -3.8 | -3.8 | - 5.0 | -7.2 |
| weight at 48 h (%) | -4.6 | -3.7 | +2.6 | +0.6 | -2.3 | -3.0 | 0.0 | -3.7 | -2.9 | -2.9 | 0 | +0.4 |

**Table S1.** Symptoms induced by MTD of novel compounds in male mice.

+ (mild), ++ (moderate), +++ (severe) symptoms.

| signs and symptoms | Tested compound | | | | | | | | | | | |
| --- | --- | --- | --- | --- | --- | --- | --- | --- | --- | --- | --- | --- |
|  | **4**  **(100 mg/kg)** | | **5**  **(200 mg/kg)** | | **6**  **(100 mg/kg)** | | **7**  **(200 mg/kg)** | | **8**  **(2000 mg/kg)** | | **10**  **(650 mg/kg)** | |
|  | 1 | 2 | 1 | 2 | 1 | 2 | 1 | 2 | 1 | 2 | 1 | 2 |
| hypersalivation |  |  |  |  |  |  |  |  |  |  |  |  |
| hyper/hypoventilation |  |  | ++ | ++ | ++ | ++ | ++ | ++ |  |  | + | + |
| exopthalmus |  |  |  |  |  |  |  |  |  |  |  |  |
| drop of eyelids | + | + | + | + | + | + |  | + |  |  |  |  |
| lacrimation |  |  |  |  |  |  |  |  |  |  |  |  |
| piloerection |  |  |  |  |  |  |  |  |  |  |  |  |
| acrocyanosis |  |  |  |  |  |  |  |  |  |  |  |  |
| ataxia |  |  | + | + | ++ | ++ |  |  |  |  | + | + |
| prostration | + | + |  |  | + | + | + | + |  |  | ++ | ++ |
| tremor |  |  |  | + |  |  | ++ | ++ | + | + | + | + |
| convulsion |  |  |  |  |  |  |  |  |  |  | + | + |
| excitation |  |  |  |  |  |  |  |  | + | + | + | + |
| saber-like tail |  |  |  |  |  |  |  |  |  |  |  |  |
| stooping |  |  |  |  | ++ | ++ |  |  |  |  |  |  |
| weight at 24 h (%) | -5.7 | -4.1 | +4.0 | -6.5 | +7.0 | +4.1 | +1.8 | +5.2 | -0.5 | +4.5 | -1.5 | -5.6 |
| weight at 48 h (%) | -7.3 | -7.9 | +4.4 | +8.1 | +8.8 | +8.2 | +5.1 | +6.1 | -2.2 | +3.5 | +4.5 | +1.0 |

**Table S2.** Symptoms induced by MTD of novel compounds in female mice.

+ (mild), ++ (moderate), +++ (severe) symptoms.

| Control * | | Glucose | | Urea | | Creatinine | | ALT | | AST | | ALP | | Amylase | |
| --- | --- | --- | --- | --- | --- | --- | --- | --- | --- | --- | --- | --- | --- | --- | --- |
|  |  | (mg/dL) | | (mg/dL) | | (µg/dL) | | (U/L) | | (U/L) | | (U/L) | | (U/L) | |
| m (mean ± 2 × SEM) | | 174 ± 18 | | 43 ± 4 | | 38 ± 7 | | 36 ± 8 | | 104 ± 18 | | 43 ± 6 | | 2227 ± 224 | |
| m (LCL – UCL) | | 60 – 287 | | 18 – 68 | | 0 – 80 | | 0 – 88 | | 0 – 218 | | 4 – 82 | | 840 – 3615 | |
| f (mean ± 2 × SEM) | | 198 ± 13 | | 46 ± 5 | | 53 ± 8 | | 23 ± 2 | | 59 ± 8 | | 90 ± 9 | | 2478 ± 119 | |
| f (LCL – UCL) | | 128 – 267 | | 18 – 75 | | 11 – 95 | | 11 – 35 | | 19 – 99 | | 44 – 136 | | 1860 – 3097 | |
| compound (MTD) |  | m | f | m | f | m | f | m | f | m | f | m | f | m | f |
| 4 (100 mg/kg) | 1 | 227 | 228 | 53 | 38 | 45 | 79 | 43 | 28 | 121 | 61 | 63 | 76 | 2464 | 2216 |
|  | 2 | 226 | 237 | 39 | 65 | 79 | 78 | 41 | 28 | 124 | 93 | 77 | 83 | 1905 | 2632 |
| 5 (200 mg/kg) | 1 | 185 | 187 | 31 | 65 | 23 | 45 | 28 | 29 | 58 | 65 | 37 | 88 | 1757 | 2531 |
|  | 2 | 175 | 192 | 33 | 58 | 21 | 22 | 29 | 22 | 59 | 44 | 38 | 80 | 2008 | 2489 |
| 6 (100 mg/kg) | 1 | 130 | 199 | 42 | 65 | 45 | 22 | 28 | 29 | 64 | 53 | 38 | 86 | 2124 | 2922 |
|  | 2 | 177 | 212 | 35 | 55 | 23 | 34 | 25 | 27 | 62 | 50 | 36 | 95 | 2086 | 2835 |
| 7 (200 mg/kg) | 1 | 244 | 241 | 35 | 40 | 23 | 57 | 19 | 22 | 48 | 52 | 78 | 92 | 2272 | 2501 |
|  | 2 | 194 | 178 | 38 | 40 | 34 | 23 | 29 | 33 | 66 | 71 | 55 | 37 | 1858 | 1496 |
| 8 (2000 mg/kg) | 1 | 202 | 171 | 41 | 35 | 57 | 79 | 26 | 20 | 65 | 78 | 71 | 121 | 2674 | 1798 |
|  | 2 | 249 | 210 | 37 | 40 | 56 | 79 | 22 | 23 | 56 | 67 | 82 | 81 | 2327 | 2455 |
| 10 (650 mg/kg) | 1 | 189 | 225 | 47 | 38 | 34 | 68 | 24 | 27 | 58 | 55 | 42 | 67 | 2212 | 1939 |
|  | 2 | 236 | 178 | 43 | 38 | 34 | 67 | 30 | 24 | 51 | 56 | 61 | 73 | 2080 | 1826 |

**Table S3.** Biochemical parameters evaluated 48 h after administering MTD of novel compounds in male (m) and female (f) BALB/c mice. * Data collected from separate in vivo experiments (18 males and 18 females). LCL and UCL – lower and upper control levels calculated as mean ± 3 × SD. No values exceeding the upper control threshold were found.
