# Supplementary material for: Substituted Piperazines as Novel Potential Radioprotective Agents
Source: Molecules. 2020 Jan 25;25(3):532. doi: 10.3390/molecules25030532 (PMC7038073; doi:10.3390/molecules25030532)
Supplement: Supplementary file 1 [file molecules-25-00532-s001.zip › Suplementary data Suppl. Scheme S1-S6.doc]

**Scheme S1.** Synthetic pathway for 7-methoxytacrine-piperazine derivatives **2** and **3**. Reactions and conditions: (i) 1-(2-aminoethyl)piperazine, phenol, 120-130 °C; (ii) 2-bromoethanol, Hunig´s base, CH_2_Cl_2_, reflux.

**Scheme S2.** Synthetic pathway for 1-(4-chloro-3-methylphenoxy)-3-(4-(2-hydroxyethyl)piperazin-1-yl)propan-2-ol (**4**). Reactions and conditions: (i) epibromohydrin, 135 °C, 2 h, 1 drop of piperidine (cat.); (ii) 1-(2-hydroxyethyl)piperazine (**1**), MeCN, reflux, 4 h.

**Scheme S3.** Synthetic pathway for 2-(4-(3-(arylamino)propyl)piperazin-1-yl)ethanol derivatives (**5-7**). Reactions and conditions: (i) aniline (**5a**) or 1-naphthylamine (**6a**) or *p*-anisidine (**7a**), MW, 15 min, 110 °C, 1-bromo-3-chloropropane, MeCN, KI; (ii) 1-(2-hydroxyethyl)piperazine (**1**), MeCN, 4 h.

**Scheme S4.** Synthetic pathway of 1.3-bis[4-(2-hydroxyethyl)piperazin-1-yl)propan-2-ol (**8**). Reaction and conditions: (i) MeOH, r.t., 48 h, 1,3-dibrompropan-2-ol.

**Scheme S5.** Synthetic pathway of 1,3-bis(phenylamino)propan-2-ol. Reaction and conditions: MW, 50 °C, 1 h, solvent free, epibromohydrine.

**Scheme S6.** Synthetic pathway of 1-(phenylamino]-3-[4-(2-hydroxyethyl)piperazin-1-yl)propan-2-ol. Reactions and conditions: (i) EtOH abs., 0 °C-r.t., 6 h, 1-bromo-3-chloropropane-2-ol; (ii) MeCN, K_2_CO_3_, 0 °C-r.t., 24 h, 1-(2-hydroxyethyl)piperazine **1.**
